# Generation Scotland participant survey on data collection

**DOI:** 10.12688/wellcomeopenres.15354.2

**Published:** 2019-12-13

**Authors:** Rachel Edwards, Archie Campbell, David Porteous

**Affiliations:** 1 Centre for Genomic and Experimental Medicine, University of Edinburgh, Edinburgh, City of Edinburgh, EH4 2XU, UK; 2 MRC Human Genetics Unit, University of Edinburgh, Edinburgh, City of Edinburgh, EH4 2XU, UK; 3 Usher Institute for Population Health Sciences and Informatics, University of Edinburgh, Edinburgh, City of Edinburgh, EH4 2XU, UK

**Keywords:** Generation Scotland, participant survey, attitudes to data collection

## Abstract

**Background: **Generation Scotland (GS) is a population and family-based study of genetic and environmental health determinants. Recruitment to the Scottish Family Health Study component of GS took place between 2006-2011. Participants were aged 18 or over and consented to genetic studies, linkage to health records and recontact. Several recontact exercises have been successfully conducted aimed at a) recruitment to embedded or partner studies and b) the collection of additional data. As the cohort matures in age, we were interested in surveying attitudes to potential new approaches to data collection and recruitment.

**Methods: **A ten-question online survey was sent to those participants who provided an email address.

**Results: **We report a high level of positive responses to encouraging relatives to participate, to remote data and sample collection and for research access to stored newborn dried blood spots.

**Conclusions: **The majority of current and prospective GS participants are likely to respond positively to future requests for remote data and sample collection.

## Introduction

The Generation Scotland: Scottish Family Health Study (hereafter GS) cohort comprises nearly 24,000 participants in around 7,000 family groups, aged 18 or over at the time of recruitment ^1^. GP research practices in Glasgow, Tayside and Aberdeen provided letters of invitation on behalf of the study team. Participants were asked to recruit at least one family member to the study. Each recruit then completed a questionnaire on medical history, personality and lifestyle, attended a clinical examination, provided biological samples (blood and urine for genetic and biomarker studies) and consented to linkage to the routine medical records collected by NHS Scotland. We also asked permission to recontact participants for follow-on studies and 98% agreed to this. Participants are able to withdraw at any time. A small number of participants have withdrawn consent to recontact, but none have fully withdrawn to date.

Recruitment in 2006–2011 was limited by the funding available, not the willingness to participate. From information given at the time of recruitment and through maternity records, we know of a large number of first and second degree relatives of current participants that would add value to the cohort if interested and able to participate (estimated as >14,000). There are also significant numbers of younger relatives who might likewise be interested to participate.

Our research to date has shown the value of the family design to address questions that are beyond the easy reach of much larger cross-sectional studies, such as UK Biobank ^2^. These include spousal and household effects as well as parent-of-origin (genetic imprinting) effects and, of course, transgenerational effects ^3– 6^.

When originally planning our recruitment to GS, we were conscious of the added value of birth data and sought to capture this where possible by recruiting in the Tayside region to co-capture members of the Walker Cohort ^7^ and, at the tail end of recruitment, the Aberdeen Children of the Nineteen Fifties (ACONF) ^8, 9^. 

Since the end of recruitment, internet access and smart phone use has increased dramatically across all ages and demographics. We have used the recontact mechanism to seek permission to contact participants by email – a third responded and agreed.

We conducted various public engagement events prior to recruitment to GS ^10– 13^. We have also collected informal and formal feedback on participant attitudes in the past through various engagement events. Here, we describe a pilot study of attitudes to new participant recruitment and new modes of data collection. For simplicity, we used the popular SurveyMonkey tool to conduct the study, posing ten questions to guide our future planning.

## Methods

Participants were recruited to GS via GP practices in Scotland and originally by letter ^1^. Email addresses were not collected at the time of recruitment but were asked for in a subsequent recontact exercise (STRADL) ^14^, with 46% responding and 36% providing an email address. All those who shared an email address were eligible to take part in this questionnaire study. No other efforts to address potential bias were taken.

Our questionnaire, created using SurveyMonkey (San Mateo, California, USA), was entitled ‘Share your thoughts on health research’, was sent out between 3 ^rd^–10 ^th^ April 2019. The introduction to the questionnaire is shown in Box 1. The survey was sent to all 7,118 GS participants for whom we had email addresses, of which 2,613 responded (34%), the majority (76%) within a week on emailing. The time taken to complete the questionnaire averaged 2 minutes. There was no repeat emailing to non-respondents.

Box 1. **‘Share your thoughts on health research’: Survey Introduction.**

**Table:** 

Survey Introduction
Share your thoughts on health research We’re carrying out health research with the people of Scotland. For the Generation Scotland project, 24,000 people from 7,000 families have already shared information about their health, personality and lifestyle with us. They have allowed us to link all this information up to their NHS medical records and collect blood samples to look at their genetic makeup. We take very great care to keep their information safe and secure. We’ve learnt a lot already about how health risks run in families and about how these risks are affected by where you were born and brought up, your schooling and your occupation. We’re planning the next stage of our research and want to hear your views. This will help us understand what matters most to you and will help us plan our future research. The survey is entirely voluntary and confidential and anonymous. It will take 5–10 minutes to complete. You don’t have to be a member of Generation Scotland to take part, or to have been involved in health research before. Before you start, it would be useful to have some basic information about you

We started by asking about their age, gender and cohort participation. Cohort participation was queried so that the same questionnaire could be used for other cohorts and/or the general public. Here, we report only on the GS participants. We asked seven questions to explore current participant views on new participant recruitment and modes of data collection ( Table 1). Questions 4–10 were formatted in the same five-option style: Definitely yes; Probably yes; Not sure; Probably no; Definitely no. We used a panel of experts in public engagement and Patient and Public Involvement panel members to check the wordage and formatting.

**Table 1.** ‘Share your thoughts on health research’: survey questions.

Question Number	Question Asked
**1**	What age are you?
**2**	What gender do you identify as?
**3**	Have you taken part in any of these health research studies? (Tick all that apply)
**4**	If asked by researchers at a University to take part in a health study like Generation Scotland, how would you answer?
**5**	If the study asked you to invite another member of your family to join too, would you be happy to do so?
**6**	If the study asked you to give details about your own health, personality, habits, and lifestyle, would you be happy to do so?
**7**	If the study asked to use blood samples left over from routine health tests, would you agree to that?
**8**	If the study involved genetic analysis, using DNA extracted from a saliva sample kit sent to your home, would you agree to take part?
**9**	If the study involved analysis, using blood extracted from a finger prick kit sent to your home, would you agree to take part?
**10**	Children born in Scotland after 1965 have heel prick blood spots (Guthrie cards) taken to test for serious but treatable conditions. The Guthrie cards have been kept in a safe place by the NHS. A study might ask for specific permission to use these stored blood spots for research. Does this sound reasonable, whether or not your own Guthrie card has been stored?

**Figure 1.**
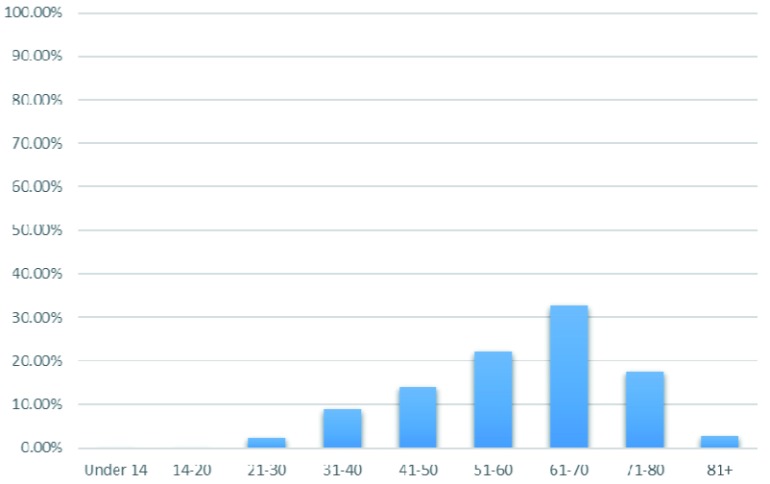
Age of recontacted participants.

**Figure 2.**
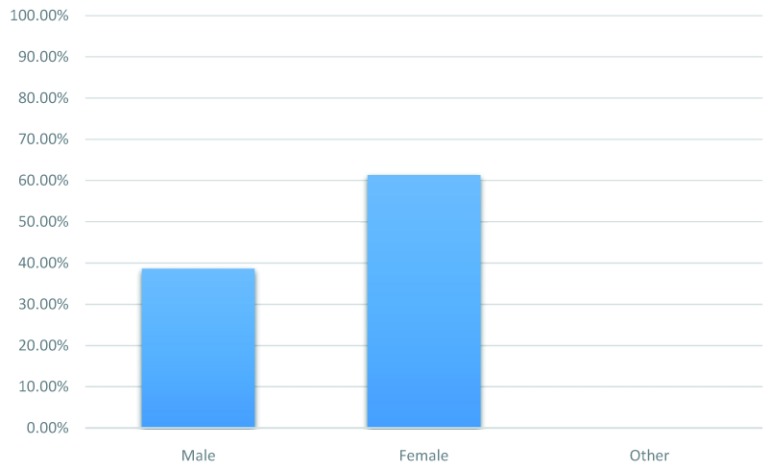
Sex of recontacted participants.

**Figure 3.**
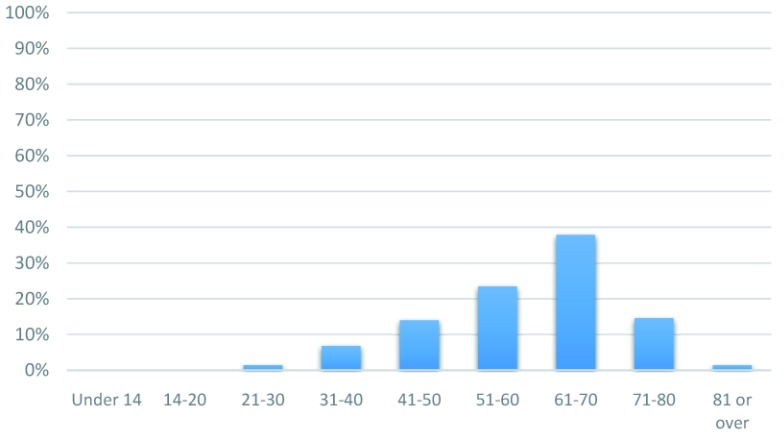
What age are you?

**Figure 4.**
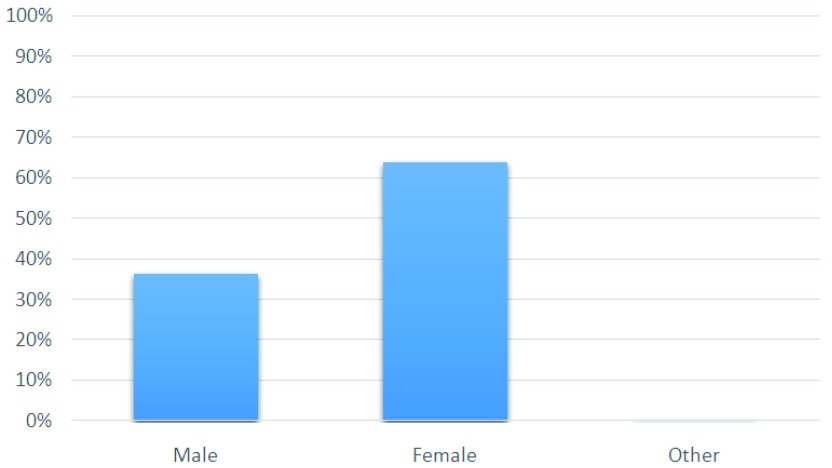
What gender do you identify as?

**Figure 5.**
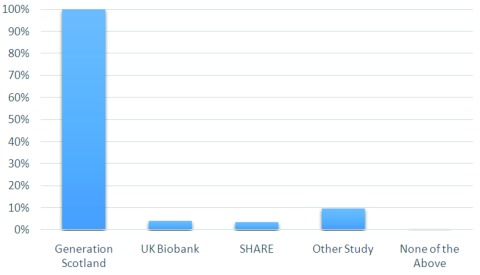
Have you taken part in any of these health research studies? (Tick all that apply).

**Figure 6.**
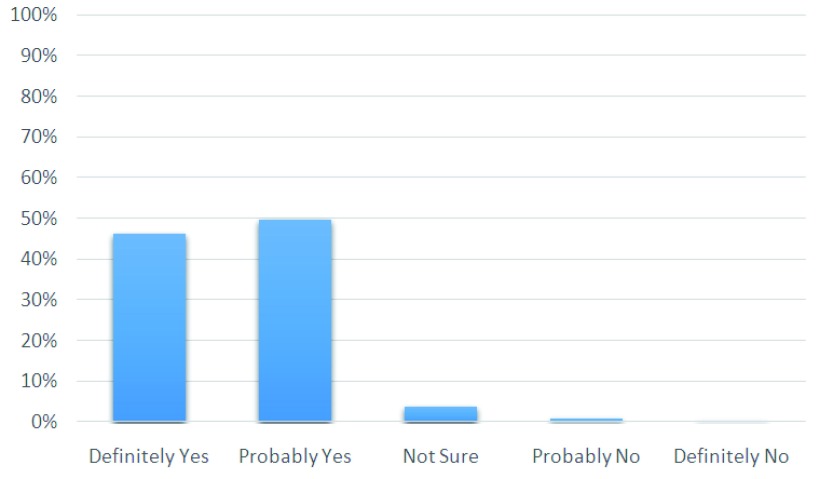
If asked by university researchers, would you join a health study like Generation Scotland?

The demographics of recontacted participants are shown in Figure 1 and Figure 2, respondent demographics in Figure 3 – Figure 5 and results of the survey questions in Figure 4, Figure 6– Figure 12. 

**Figure 7.**
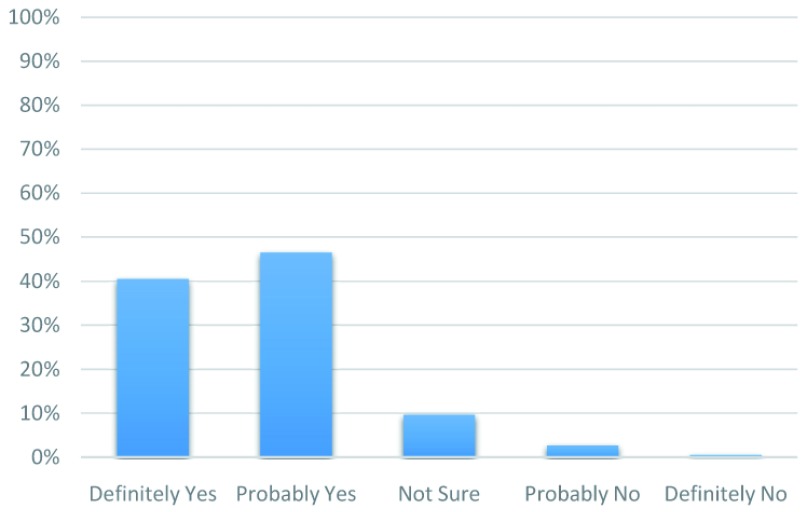
Would you invite a family member to join the health study too, if asked?

**Figure 8.**
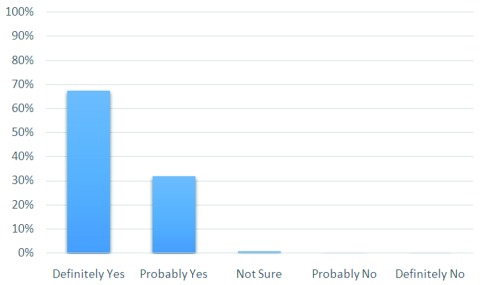
If the study asked you to answer health and lifestyle questions, would you answer?

**Figure 9.**
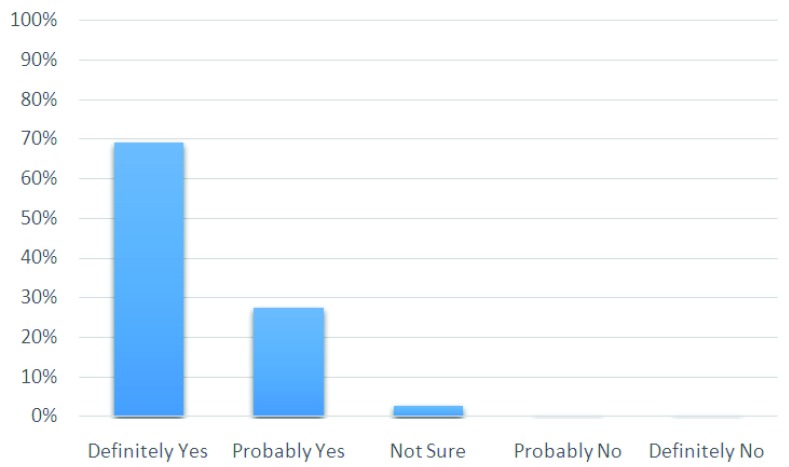
Would you agree to the study using left over blood samples from routine health tests?

**Figure 10.**
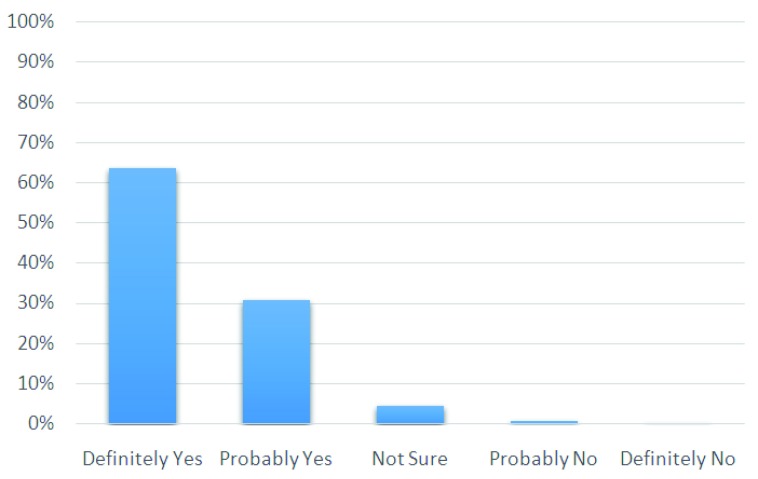
Would you agree to join if asked to provide a saliva sample for genetic analysis?

**Figure 11.**
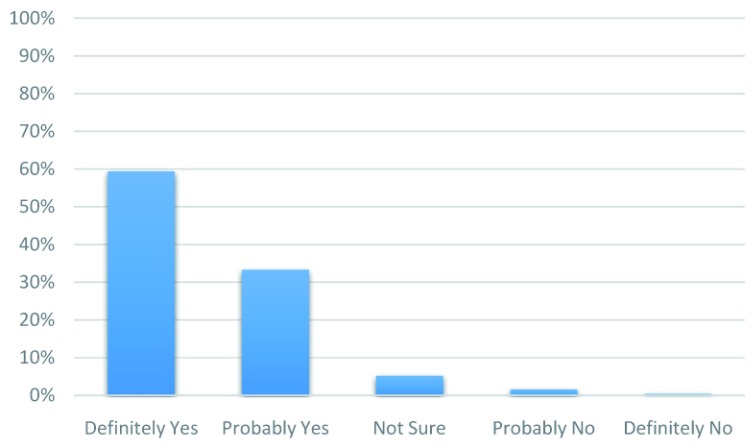
Would you take part if asked to complete a home finger prick blood test?

**Figure 12.**
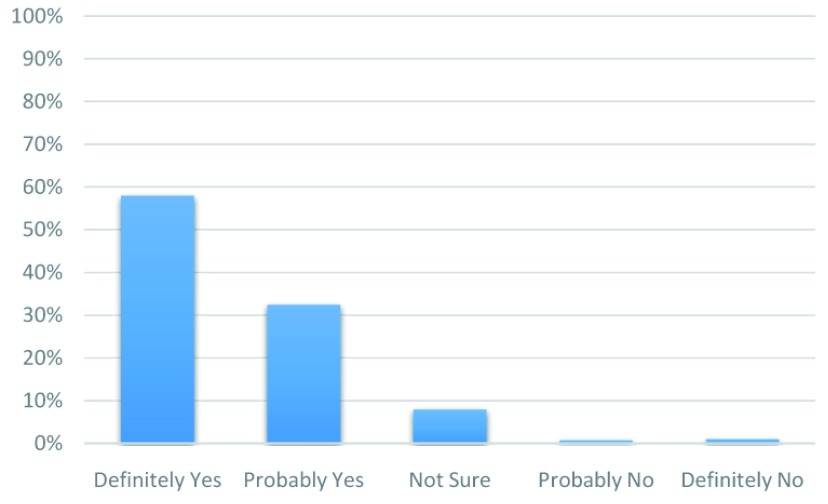
Would you agree to your Guthrie card being used for analysis?

### Ethical issues

Generation Scotland is an approved research tissue bank. It was approved by East of Scotland Research Ethics Service (EoSRES) under reference LR/15/ES/0040. GS participants consented to recontact when they joined the Generation Scotland study. Participants who previously provided an email address were invited to take part via email, with participation in the survey being entirely voluntary. GS participants are free to withdraw at any time.

## Results and discussion

The results of this Survey Monkey are completely anonymous, so it is not possible to provide a breakdown of how different individuals, age groups or sexes responded. There were however more female (1,660, 64%) than male (944, 36%) respondents, but this reflects in part the baseline participation (59% female:41% male) and those sharing email addresses (61% female, 39% male). The age range of responders closely matched that of those contacted ( Figure 1 and Figure 3). Respondents were consistently positive in answers to questions 4–10, with over 80% replying ‘definitely yes’ or ‘probably yes’. The definite ‘no’ category was returned by 1% or less of respondents. Individual and summary level results of the survey are available as *Underlying data* ^15^.

The questions that sought opinions on the acceptability of potential future means of recruitment, data and sample collection are shown in Table 1. Question 4 was posed because the original recruitment was by a letter of introduction by their GP. It was encouraging that recruitment via researchers at a University met with a strongly positive response. Question 5 was posed because our GS research to date has highlighted the value of family structure, and both genetic and non-genetic (spousal, household, environmental) effects on health and health trajectories ^3– 6^. Question 6 was posed because this type of information was included in the original pre-clinic questionnaire and has been of great value in addressing questions relating to mental health, cognition, personality and lifestyle on physical health, and vice versa ^3, 4, 6, 16, 17^. Question 7 was posed because this could be a very cost-effective way of collecting blood samples (and potentially other left-over pathology samples). In Scotland, the SHARE mechanism is already in place to consent to left-over blood being made available for research ^18^. Over 250,000 have already signed up at https://www.registerforshare.org/. Questions 8 and 9 were posed because clinic-based (or GP practice based) recruitment is costly and can exclude or limit participation for geographical reasons. By contrast, online shopping and postal delivery of a wide range of products, including medical products and prescriptions, is now commonplace. Home-based kits for saliva and blood samples are well tried and tested. Blood prick sampling has been the norm for blood glucose measurements in diabetes for many years and is well tolerated.

Question 10 sought opinions on research access to archived new-born dried blood spots. Heel prick blood sampling in new-borns is standard practice in many countries and used to screen for a well-defined set of rare and preventable metabolic disorders and/or detection of genetically inherited conditions that benefit from early detection. These dried blood spots can be used for a variety of other tests, including infection, toxins and epigenetic modification (DNA methylation) ^19, 20^. In many countries, these cards are held long-term. In Scotland, new-born heel prick dried blood samples (historically referred to as Guthrie cards) have been collected and retained since 1965, 3 million to date, increasing by around 60,000 per annum. We estimate that up to 9,700 (40%) of current GS participants will have a blood spot card retained and stored securely by the NHS Scotland National Screening Service. In some countries, such as Denmark, research access is possible under agreed conditions and for agreed purposes ^15^. Whilst this is not currently approved in the UK, we felt it important to test current opinion amongst medical research participants. Although 1% of respondents said definitely ‘no’ to research use of their stored dried blood spot card on principle, over 90% said definitely or probably ‘yes’.

In summary, we found that there was a very positive overall response to questions designed to assess the likely ‘buy in’ to a cost-effective extension to the Generation Scotland cohort. The high response rate suggested that this easy-to-offer approach could be a useful tool for further engagement and shaping of cohort retention, expansion and enhancement. One caveat to the wider interpretation of our results is that we are consulting existing participants, not the general public or hard-to-reach constituents. Further comparative contact studies are warranted. For the practical purpose however of scoping the possible expansion and extension of GS, these findings are valuable and encouraging.

## Data availability

The SurveyMonkey study was by pseudo-anonymised email recontact. All summary and individual level survey responses to this can be found in DataShare.

### Underlying data

Edinburgh DataShare: Generation Scotland Survey Monkey data. https://doi.org/10.7488/ds/2585 ^15^. This project contains the following underlying data:

Generation Scotland Survey Monkey data.xlsx (includes all summary and individual level data collected in the survey).

Data are available under the terms of the Creative Commons Attribution 4.0 International license (CC-BY 4.0).

Researchers can request access to this and all other GS data by contacting the GS Access Committee. A phenotype data dictionary is available and open access GWAS summary statistics can be downloaded. Non-identifiable information from the GS:SFHS cohort is available to researchers in the UK and to international collaborators through application to the GS Access Committee. GS operates a managed data access process including an online application form, and proposals are reviewed by the GS Access Committee. Summary information to help researchers assess the feasibility and statistical power of a proposed project is available on request by contacting resources@generationscotland.org.
