# Supplementary material for: Generation Scotland participant survey on data collection
Source: Wellcome Open Res. 2019 Dec 13;4:111. Originally published 2019 Jul 25. [Version 2] doi: 10.12688/wellcomeopenres.15354.2 (PMC6964360; doi:10.12688/wellcomeopenres.15354.2)
Supplement: Peer review history

# Peer review: Reviewer response for version 2

**DOI:** 10.21956/wellcomeopenres.17013.r37387

**Published:** 2020-01-15

**Authors:** Claire J. Steves

**Affiliations:** 1 The Department of Twin Research and Genetic Epidemiology, St Thomas’ Hospital, Kings College London, London, UK

I’m happy to approve. No additional comments.

I confirm that I have read this submission and believe that I have an appropriate level of expertise to confirm that it is of an acceptable scientific standard.

# Peer review: Reviewer response for version 1

**DOI:** 10.21956/wellcomeopenres.16769.r36432

**Published:** 2019-11-26

**Authors:** Claire J. Steves

**Affiliations:** 1 The Department of Twin Research and Genetic Epidemiology, St Thomas’ Hospital, Kings College London, London, UK

This is a clearly written report surveying 36% of Generation Scotland participants who have given their email addresses on their views on extension of the study. The response rate was 34%. Generally the responses are very positive about planned initiatives. I would be keen for the report to also include information (e.g. a table) on the demographic characteristics of the non-responders, and those who did not have or did not share their email, to assess what bias either of these sub-setting processes might have had. It is likely that responders may be slightly more likely to engage with the initiatives planned. Provided the survey responses can be linked to the demographics of the participants collected at enrolment, more detail on the characteristics of the ~1% less willing subjects, together with the numbers with these characteristics in the whole cohort could be informative of the bias which might occur if consent for these initiatives is sought.

I confirm that I have read this submission and believe that I have an appropriate level of expertise to confirm that it is of an acceptable scientific standard, however I have significant reservations, as outlined above.

# Peer review: Reviewer response for version 1

**DOI:** 10.21956/wellcomeopenres.16769.r36433

**Published:** 2019-10-07

**Authors:** Elaine Douglas

**Affiliations:** 1 University of Stirling, Stirling, UK

Scotland has a rich data infrastructure which makes it well placed to conduct biomedical research that can be linked to individual survey results and administrative data. The potential of such infrastructure can only be achieved by the trust and consent of the public to take part in such research. This paper provides a welcome overview of the willingness of participants to take part in such research. I recommend this paper for indexing. 

 Comments

- While the sample are drawn from existing GS participants and therefore not generalisable to the wider population per se, the response mode and response rates are relevant, and will be of interest to, other researchers who may be considering a follow-up to their own participants.
- The results of this research e.g. the willingness of the sample to take part in health studies, to support recruitment via family members, and to give consent to use of blood samples, etc. is of value to researchers working within this field, and gives some insight (with the aforementioned caveats) of the acceptability of such research and recruitment methods.

Suggestions:

- If the y-axis of all figures were produced using the same scale this would ease the comparison of responses across all questions.

I confirm that I have read this submission and believe that I have an appropriate level of expertise to confirm that it is of an acceptable scientific standard.

# Response

**Published:** 2019-10-22

**Authors:** David Porteous

**Affiliations:** University of Edinburgh, UK

Thank you for these comments. We will provide a revised submission with all of the questionnaire responses on the same scale. We have not changed any of the text.
